# Age‐Specific Associations of Polygenic Risk Scores With Advanced Fibrosis and Histological Activity in Biopsy‐Proven MASLD

**DOI:** 10.1111/liv.70907

**Published:** 2026-10-01

**Authors:** Samer Gawrieh, Jingyi Tan, Xiuqing Guo, Linus Schwantes‐An, Marco Abreu, Callie J. Zaborenko, Eduardo Vilar‐Gomez, Jeffrey B. Schwimmer, Jerome I. Rotter, Naga Chalasani

**Affiliations:** ^1^ Division of Gastroenterology and Hepatology Indiana University School of Medicine Indianapolis Indiana USA; ^2^ The Institute for Translational Genomics and Population Sciences, Department of Pediatrics The Lundquist Institute for Biomedical Innovation at Harbor‐UCLA Medical Center Torrance California USA; ^3^ Department of Medical and Molecular Genetics Indiana University School of Medicine Indianapolis Indiana USA; ^4^ Division of Gastroenterology, Hepatology, and Nutrition, Department of Pediatrics University of California San Diego School of Medicine La Jolla California USA; ^5^ Department of Gastroenterology Rady Children's Hospital San Diego San Diego California USA

**Keywords:** at risk, gene, genetic, MASH, personalized medicine

## Abstract

**Background and Aims:**

The genetic susceptibility to MASLD histological severity remains incompletely defined. We assessed the associations between recently developed genome‐wide association studies–derived polygenic risk scores (PRSs) and advanced fibrosis in MASLD. We also evaluated PRSs' associations with histological activity and selected PRSs' predictive ability for advanced fibrosis.

**Methods:**

We analysed 2149 adults and 900 children with biopsy‐proven MASLD from the NASH Clinical Research Network studies. Genotyping was performed by the Regeneron Genetics Center using whole‐exome sequencing with targeted genotyping. Associations between seven published PRSs (Chen, Emdin, Whitfield, Schwantes‐An, Vujkovic, and Ghouse) and MASLD histological features were examined using linear and logistic regression models. Penalized regression models were used to select top PRSs associated with MASLD traits.

**Results:**

The Schwantes‐An PRS in adults (OR 1.29, 95% CI 1.17–1.42, *p* < 0.01) and the Chen PRS in children (OR 1.46, 95% CI 1.17–1.82, *p* < 0.01) were associated with higher risk of advanced fibrosis versus other PRSs. The Chen PRS in adults (OR 1.29, 95% CI 1.18–1.41, *p* < 0.01) and Schwantes‐An PRS in children (OR 1.39, 95% CI 1.20–1.61, *p* < 0.05) were associated with higher risk with MASLD activity. Although highest PRSs quartiles were associated with advanced fibrosis risk versus bottom quartiles, neither PRS had good diagnostic discrimination for advanced fibrosis (AUROC < 60%).

**Conclusions:**

In this large biopsy‐proven MASLD cohort, two distinct PRSs were associated with disease histological severity and activity in adults versus children. Findings suggest current PRSs may improve personalized risk stratification for advanced fibrosis but not its diagnosis.

## Key Points

- MASLD is a complex disease with multiple genetic, metabolic, and environmental factors influencing its risk and severity.
- In this large biopsy‐proven MASLD cohort, two distinct polygenic risk scores (PRSs) were associated with disease histological severity and activity in adults versus children.
- Neither PRS had good diagnostic discrimination for advanced fibrosis or NAS ≥ 4 (AUROC < 60%).
- Findings suggest current PRSs may improve personalized risk stratification for advanced fibrosis but not its diagnosis.

## Lay Summary

- MASLD is a complex disease with multiple genetic, metabolic, and environmental factors influencing its risk and severity.
- Polygenic risk scores capture the effects of individual gene variants on a person's lifetime risk for a given trait.
- In this large study, two polygenic risk scores were significantly associated with MASLD activity and severity both in children and adults.

## 1 Introduction

Metabolic dysfunction‐associated steatotic liver disease (MASLD) is the most common liver disease in the US, affecting an estimated 10% of children and 30% of adults [1, 2]. MASLD histological activity and severity drive clinical outcomes. Patients with the mild phenotype, simple steatosis, rarely progress, whereas those with the more histologically active phenotype, metabolic dysfunction‐associated steatohepatitis (MASH), have higher rates of progression to cirrhosis, liver failure, and cancer [3, 4]. MASLD disease histological activity is associated with the development and progression of hepatic fibrosis, which in turn is the strongest predictor of future liver‐related outcomes [5, 6]. In this context, patients with MASLD who combine histologically active disease with significant fibrosis are at a particularly increased risk for progression to cirrhosis and poor outcomes, and thus represent a high priority for effective interventions [7, 8].

The genetic susceptibility to developing histologically active and severe MASLD remains incompletely understood. The discovery of the patatin‐like phospholipase domain‐containing protein 3 (*PNPLA3*) variant as a modifier of MASLD risk, severity, and subsequent clinical outcomes ushered in a new era in MASLD genetics [9, 10, 11, 12]. Subsequently, multiple other genetic variants were discovered and shown alone or in combination with other candidate gene variants to be associated with susceptibility to or protection from the development of MASLD, its histological activity and severity, or its complications [13, 14, 15, 16, 17, 18, 19, 20].

To better estimate an individual's risk of developing MASLD, genome‐wide association studies (GWAS)‐derived polygenic risk scores (PRSs) have been investigated [21, 22]. PRSs capture the combined weighted effects of individual genetic variants on the risk of a trait [23], and may therefore facilitate a more refined risk stratification and personalized approach to diagnosis and management depending on identified risk level. Predicting early in life which individuals are at high genetic risk for developing severe progressive MASLD may allow earlier diagnosis, followed by lifestyle and pharmacological interventions to prevent development of end‐stage liver disease. Further, for individuals with established MASLD, those identified to be at high genetic risk of developing liver cirrhosis and cancer could be followed and surveyed more closely for these outcomes [24].

Beyond association with histologically proven MASLD [21], the associations of recently described GWAS‐derived PRSs with the histological activity and severity of MASLD have not been examined in adults or children with MASLD.

Our primary aim was to assess the association of recent GWAS‐derived PRSs with advanced fibrosis in a large prospective cohort of adults and children with histologically characterized MASLD. Our secondary aims were to assess the association of these PRSs with MASLD histological activity and identify the best PRSs that predict the risk of advanced fibrosis and high histological activity.

## 2 Methods

### 2.1 The Study Cohort

This study included data from adults and children enrolled in the Nonalcoholic Steatohepatitis Clinical Research Network (NASH CRN) studies. This prospective cohort has been described previously [25, 26]. Briefly, patients with biopsy‐confirmed MASLD were prospectively recruited at multiple centers across the United States between 2004 and 2020. Patients were included in this cohort based on the presence of MASLD. The diagnosis of MASLD was based on the presence of steatosis (≥ 5% of hepatocytes containing macrovesicular steatosis) and exclusion of significant alcohol consumption (average of > 20 g daily for women, > 30 g daily for men) within 2 years of the initial biopsy. Each participant or their legally authorized representative provided informed consent to participate in the parent NASH CRN study.

### 2.2 Histological Phenotyping and Selection of Primary Phenotypes for Association Analyses

All liver biopsies were reviewed in a blinded fashion by the NASH CRN Pathology Committee and scored according to the NASH CRN Scoring System [27]. Fibrosis stage was assessed from 0 (no fibrosis) to 4 (cirrhosis). Advanced Fibrosis was defined as Stage 3 and 4 (F ≥ 3) fibrosis. Steatosis was graded from 0 to 3, hepatocellular ballooning from 0 to 2, and lobular inflammation from 0 to 3; the presence of these three lesions was essential for the diagnosis of definite MASH and the sum of them reflected the non‐alcoholic fatty liver disease activity score (NAS) [27]. At‐risk MASH was defined as MASH with NAS ≥ 4 and stage ≥ 2 fibrosis [28]. Fibrosis was selected as the primary histological trait for the association studies since it reflected MASLD histological severity, which impacts MASLD clinical course and outcomes [3, 4, 5, 6]. Secondary analyses were conducted to evaluate the association of PRSs with NAS, MASH cardinal lesions (steatosis, ballooning, and lobular inflammation), and at‐risk MASH.

### 2.3 Genotyping Procedures

Those have been described previously [29]. Briefly, the NASH CRN established a research collaboration with the Regeneron Genetics Center (RGC) through an ancillary study which was approved by the NASH CRN Steering Committee. This collaboration follows the National Institutes of Health (NIH) Genomic Data Sharing policies and procedures. As part of this collaboration, the RGC conducted whole‐exome sequencing (WES) at no cost to the NASH CRN and returned results to the NASH CRN. An analytical team established by the NASH CRN team at Indiana University conducted analyses described in this paper.

Genotype data were generated by RGC using RGC's WES plus targeted genotyping assay. DNA samples were sequenced for 20× targeted WES and over 1.4 million targeted variants. Genotype data were cleaned using our previously published data QC pipeline [30]. Briefly, we removed samples and variants with less than 95% call rates and also samples with discordant reported and genetic sex. Expected and unexpected relationships between samples were evaluated. SNPsRelate was used to cluster NASH‐CRN samples with African (AFR), Amerindian American or Hispanic (AMR), and European (EUR) ancestry groups from the 1000 Genome project (1KGP) [31, 32]. A stratified check of genetic ancestry for Hardy–Weinberg Equilibrium (HWE) was done to remove those variants that deviated from HWE in any of the three groups. Quality control passing genotype data was then imputed to TOPMED reference panel using the TOPMED Imputation Server [33]. We used *R* ^2^ of ≥ 0.8 as a measure of imputed genotype quality and checked for violation of HWE.

### 2.4 Polygenic Risk Scores Selection, Calculations and Correlations

The association of candidate gene variants with MASLD histology was previously assessed in children and adults included in this cohort [29, 34]. For this analysis, we selected recently reported PRSs derived from large GWAS studies which demonstrated association with the risk of MASLD or cirrhosis. Two PRSs associated with the risk of alcohol‐associated liver disease (ALD) cirrhosis were also included based on known shared genetic risks between MASLD and ALD [35, 36]. Selection of the PRSs included in this analysis was finalized in December 2024.

PRSs were constructed based on recent publications by Chen et al. [17 Single Nucleotide Polymorphisms (SNPs)] [22], Emdin et al. (12 SNPs) [37], Whitfield et al. (3 SNPs) [38], Schwantes‐An et al. (20 SNPs) [39], Vujkovic et al. (77 SNPs) [21], and Ghouse et al. [40] (36 SNPs). Each PRS was constructed by summing the products of allele count (dosage) at each SNP multiplied by their reported effect sizes over all loci. SNPs absent from the TOPMed imputed dataset or failing post‐imputation QC were excluded from the computation. In addition to their 77 SNP PRS which was developed to predict biochemically‐defined MASLD [21], Vujkovic et al. also replicated a PRS consisting of 17 out of the 77 SNPs that was associated with histologically‐ and/or radiologically‐defined MASLD (Vujkovic 17‐SNPs PRS) [21]. We also evaluated this PRS in the current analysis (Table S1). All SNPs were available for three PRSs, whereas four of the PRSs had ≤ 3 missing SNPs (Table S2).

To examine the distribution and interrelationships of the PRSs, histograms of the raw PRSs values were generated, and Pearson correlation coefficients were calculated among all scores. The value for the strength of the correlation between the PRSs was interpreted using the guide suggested by Evans as follows: 0.00–0.19 “very weak”, 0.20–0.39 “weak”, 0.40–0.59 “moderate”, 0.60–0.79 “strong”, 0.80–1.00 “very strong” [41]. PRSs values were standardized within each ancestry group to have a mean of 0 and a standard deviation of 1.

### 2.5 Analyses of PRSs Associations With MASLD Histological Phenotypes

The standardized PRSs were used for the following association analyses to examine the relationship between PRSs and MASLD histological phenotypes. Generalized linear regression models were used for continuous outcomes, including fibrosis stage and NAS, where the effect size of a PRS association with the trait means amount of change in the trait per standard deviation change in the PRS; for example, a PRS effect size of 0.15 means a 0.15 increase in fibrosis (on a scale from 0 to 4) or a 0.15 increase in NAS (on a scale from 0 to 8). Logistic regression models were applied to binary outcomes, including advanced fibrosis (F ≥ 3), NAS ≥ 4, at‐risk MASH, steatosis Grade ≥ 2, lobular inflammation Grade ≥ 2, and ballooning Grade ≥ 1. To increase the analysis power, we combined the two majority populations in the study cohort, participants of AMR (Hispanic) and EUR genetic ancestry, adjusting for ancestry, sex, and age as covariates. Adults and children were analysed separately.

To evaluate the relative contribution of each PRS for F ≥ 3 and NAS ≥ 4 and to rank the PRSs' association with these two histological phenotypes, we employed forward stepwise model selection based on the Akaike Information Criterion (AIC). At each step, the PRS which led to the greatest reduction in AIC was added to the model. This process continued iteratively until no additional PRS would improve the model fit. Only PRSs that meaningfully contributed to model performance based on AIC were retained in the final multivariable models. To assess the stability of PRS selection in the presence of correlated PRSs, we employed penalized regression of additional models—LASSO, elastic‐net regression, and ridge, including all PRSs simultaneously. We performed cross‐validation of the results by repeated 5‐fold cross‐validation (100 repeats), ranked the overall contribution via absolute value of the coefficients, calculated the frequency of each PRS selected in the final model, calculated top contributor frequency in (EUR + AMR) samples, and analysed adults and children separately after adjusting for ancestry, age, and sex.

Given potential confounding that may be caused by demographic differences between the age groups and to evaluate whether observed findings may reflect shared genetic information or broader cohort‐level differences rather than age‐specific PRS biological effects, we performed sensitivity analyses of associations of the PRSs with the selected phenotypes stratified by ancestry using dominant ancestry subgroups of each age group (EUR in adults, *n* = 1784 and AMR in children, *n* = 681). These sensitivity analyses were not performed in the other racial subgroups in each age group due to smaller numbers of participants (adult AMR *n* = 356, and children EUR *n* = 218) not allowing adequate power for these analyses. We then examined interactions between age and sex and model‐selected top PRSs.

We assessed associations between PRS quartiles and advanced fibrosis, NAS ≥ 4, at‐risk MASH, and MASH cardinal histological features (steatosis, lobular inflammation, and ballooning) using logistic regression models, with the lowest quartile group as the reference.

To address the potential clinical utility for risk stratification, we assessed the predictive performance of PRSs by reporting the area under the receiver operating characteristic curve (AUROC) for detecting advanced fibrosis and NAS ≥ 4 in adults and children in comparison to the most commonly used non‐invasive test, FIB‐4. Next, we evaluated the additive value of combining FIB‐4 and PRSs with or without additional clinical variables such as age, sex, ancestry, obesity, and diabetes on the discriminative function of these measures for advanced fibrosis and NAS ≥ 4. To do that, we constructed and compared seven models: Model 1: FIB‐4, Model 2: PRS, Model 3: FIB4 + demographic covariates (ancestry + age + sex), Model 4: PRS + demographic covariates, Model 5: FIB4 + PRS + demographic covariates, Model 6: FIB4 + Type 2 diabetes + obesity + demographic covariates, Model 7: FIB4 + PRS + Type 2 diabetes + obesity + demographic covariates. We ran adults and children separately in the entire cohort (EUR + AMR) after adjusting for ancestry, age, and sex. DeLong's test was used to compare the differences between models' AUROC.

All statistical analyses were performed using R version 4.2.1. A two‐sided *p*‐value < 0.05 was considered statistically significant.

## 3 Results

### 3.1 Participants Characteristics

As shown in Table S3, 2149 adults and 900 children with MASLD were included in the analysis. Adults, compared to children, were more likely to be females (63% vs. 27%), White (83% vs. 24%), non‐Hispanic (83% vs. 24%), and had larger waist circumference (110 vs. 104 cm), higher BMI (34.6 vs. 32.1 kgm^2^), higher frequency of obesity (74% vs. 59%), and T2DM (37% vs. 4%). The distribution of MASLD histological phenotypes is shown in Table S3. The mean NAS was 4.32 in adults and 4.07 in children. Adults had a higher frequency of NAS ≥ 4 (67% vs. 64%), definite MASH (57% vs. 22%), at‐risk MASH (40% vs. 21%), and advanced fibrosis (31% vs. 14%) than children. The distribution of each of the PRSs in the cohort is shown in Figure S1. Overall, children had slightly higher median PRSs compared to adults.

### 3.2 Correlation Between the Seven PRSs

Overall, there were strong to very strong correlations (*r* > 0.59) among the PRSs tested except for the Vujkovic 77‐SNPs PRS, which moderately correlated (*r* = 0.44–0.59) with other scores with the expected exception of strong correlation with its abbreviated version, Vujkovic 17‐SNPs PRS (Figure 1).

**FIGURE 1:**
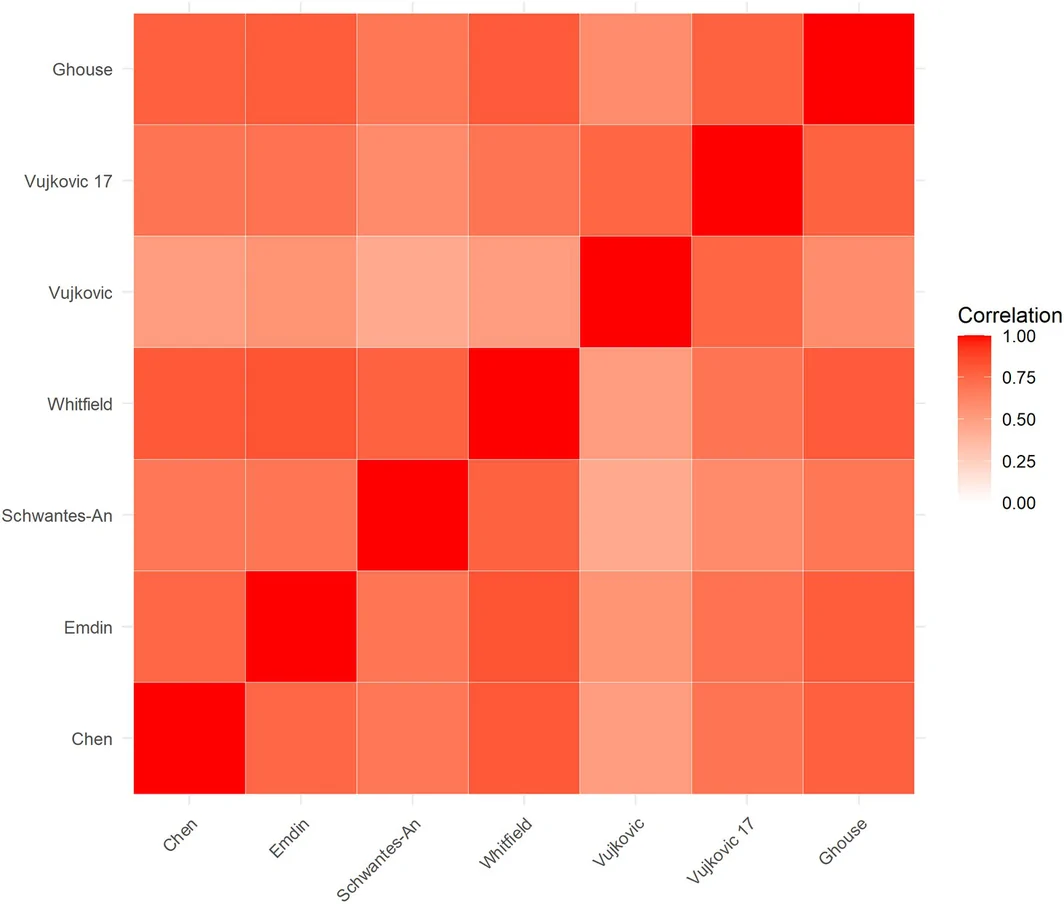
Correlation between the polygenic risk scores. Darker red colour indicates stronger correlation. Overall, there were strong correlations (*r* > 0.59) among the PRSs tested except for the Vujkovic 77‐SNPs PRS.

### 3.3 Association of PRSs With Hepatic Fibrosis

In adults, the Chen, Emdin and Ghouse PRSs had larger effect size than other PRSs on fibrosis as a continuous trait (all three PRSs had the same effect size: 0.17, and same SE: 0.03) (Table 1). The Schwantes‐An PRS was associated with higher risk of advanced fibrosis as a binary trait (≥ vs. < F3) (OR: 1.29, 95% CI: 1.17, 1.42), followed by Emdin PRS (OR: 1.28, 95% CI: 1.16, 1.41) and Whitfield PRS (OR: 1.24, 95% CI: 1.12, 1.36, *p* < 0.001 for all associations) (Table 2).

**TABLE 1:** Association of polygenic risk scores with fibrosis and MASLD activity as continuous traits.

PRS	Trait	Adult			Children		
		Effect size	Standard error	*p*	Effect size	Standard error	*p*
Chen	Fibrosis (0–4)	0.17	0.03	6.81E‐10	0.14	0.04	1.21E‐04
Schwantes‐An		0.16	0.03	6.83E‐09	0.1	0.04	5.17E‐03
Emdin		0.17	0.03	2.09E‐10	0.09	0.04	1.52E‐02
Vujkovic 77 SNPs		0.08	0.03	2.71E‐03	0.07	0.03	3.88E‐02
Whitfield		0.16	0.03	4.58E‐09	0.09	0.04	1.87E‐02
Vujkovic 17‐SNPs		0.16	0.03	4.66E‐09	0.11	0.03	1.31E‐03
Ghouse		0.17	0.03	3.55E‐10	0.12	0.04	7.46E‐04
Chen	NAS (0–8)	0.23	0.04	9.15E‐11	0.23	0.05	2.28E‐05
Schwantes‐An		0.18	0.04	4.53E‐07	0.2	0.05	1.30E‐04
Emdin		0.17	0.04	4.53E‐06	0.17	0.05	1.55E‐03
Vujkovic 77‐SNPs		0.13	0.04	5.39E‐04	0.18	0.05	3.28E‐04
Whitfield		0.19	0.04	2.14E‐07	0.15	0.05	4.58E‐03
Vujkovic 17‐SNPs		0.18	0.04	8.25E‐07	0.19	0.05	2.19E‐04
Ghouse		0.17	0.04	2.63E‐06	0.18	0.05	9.99E‐04

*Note:* Data shown for adults and children of European and Amerindian American (Hispanic) ancestry and adjusted for ancestry, age, and sex.

Abbreviation: NAS, non‐alcoholic fatty liver disease activity score.

**TABLE 2:** Association of polygenic risk scores with NAS and fibrosis as categorical traits.

PRS	Trait	Adult			Children		
		Odds ratio	95% CI	*p*	Odds ratio	95% CI	*p*
Chen	Advanced Fibrosis	1.23	(1.12–1.35)	2.25E‐05	1.46	(1.17–1.82)	8.02E‐04
Schwantes‐An		1.29	(1.17–1.42)	2.81E‐07	1.29	(1.05–1.59)	1.76E‐02
Emdin		1.28	(1.16–1.41)	4.43E‐07	1.28	(1.04–1.57)	2.14E‐02
Vujkovic		1.08	(0.99–1.19)	9.81E‐02	1.1	(0.91–1.34)	3.14E‐01
Whitfield		1.24	(1.12–1.36)	1.40E‐05	1.26	(1.02–1.56)	3.47E‐02
Vujkovic17		1.21	(1.10–1.34)	7.98E‐05	1.3	(1.06–1.59)	1.09E‐02
Ghouse		1.23	(1.12–1.36)	1.92E‐05	1.36	(1.10–1.68)	4.13E‐03
Chen	NAS ≥ 4	1.29	(1.18–1.41)	5.69E‐08	1.37	(1.18–1.59)	5.12E‐05
Schwantes‐An		1.2	(1.10–1.31)	8.76E‐05	1.39	(1.20–1.61)	1.29E‐05
Emdin		1.22	(1.11–1.34)	2.09E‐05	1.28	(1.11–1.49)	9.89E‐04
Vujkovic		1.13	(1.03–1.24)	9.87E‐03	1.3	(1.13–1.49)	2.60E‐04
Whitfield		1.23	(1.12–1.35)	9.68E‐06	1.24	(1.07–1.44)	3.75E‐03
Vujkovic17		1.25	(1.14–1.37)	3.20E‐06	1.26	(1.10–1.46)	1.21E‐03
Ghouse		1.2	(1.09–1.31)	1.12E‐04	1.26	(1.09–1.46)	2.29E‐03

*Note:* Data shown for adults and children of European and Amerindian American (Hispanic)and adjusted for ancestry, age, and sex.

Abbreviation: NAS, non‐alcoholic fatty liver disease activity score.

In children, the Chen PRS had a larger effect size on the risk of fibrosis as a continuous trait (effect size: 0.14, SE: 0.04) and was associated with a higher risk of fibrosis as a binary trait (≥ vs. < F3) (OR: 1.46, 95% CI: 1.17, 1.82) compared to other PRSs (Tables 1 and 2, *p* < 0.001 for all associations).

In a sensitivity analysis, we observed an interaction for sex with the Chen, Emdin, and Vujkovic 17 PRSs on the risk of advanced fibrosis only in EUR (Table S4).

### 3.4 Association of PRSs With MASLD Activity

In adults, the Chen PRS had the largest effect size on NAS as a continuous trait (effect size: 0.23, SE: 0.04) and was associated with higher risk of NAS as a binary trait (≥ vs. < 4) (OR: 1.29, 95% CI: 1.18, 1.41) compared to other PRSs (Tables 1 and 2).

In children, the Chen PRS had the strongest association with NAS as a continuous variable (effect size: 0.23, SE: 0.05) (Table 1), but when NAS was treated as a binary variable (Table 2), the strength of association with the Chen PRS (OR: 1.37, 95% CI: 1.18, 1.59) followed that of the Schwantes‐An PRS (OR: 1.39, 95% CI: 1.20, 1.61) (*p* < 0.001 for all associations). In a sensitivity analysis, we observed no interaction for age or sex with PRS for the risk of NAS ≥ 4 in EUR or AMR (Table S5).

### 3.5 Selection of PRSs Associated With Fibrosis and NAS for Further Association Analyses

Analyses using penalized regression of several selection models and simultaneously including all PRSs showed age‐specific selection of PRS for each trait (Table 3). The Schwantes‐An and Emdin's emerged as the leading PRSs for advanced fibrosis in adults, with the Schwantes‐An PRS being selected by most penalized approaches. The Chen PRS was more frequently selected by models as the lead PRS associated with advanced fibrosis in children than other PRSs. The Chen PRS in adults and Schwantes‐An PRS in children were more frequently selected by models as the lead PRSs associated with NAS ≥ 4 compared to other PRSs. Sensitivity analysis showed the stability of PRS selection in the dominant racial subgroup in each age group (Table S6). Therefore, further analyses were carried out exploring the associations of these two PRS with other key MASLD phenotypes.

**TABLE 3:** Frequency for the top selected PRS for advanced fibrosis and NAS ≥ 4 in penalized regression models including all PRSs simultaneously^a^.

Trait/Group/PRS	Stepwise AIC	LASSO	Elastic‐Net	Ridge
Advanced fibrosis				
Adult (*n* = 2140)				
Schwantes‐An PRS	0.444	0.532	0.534	0.52
Emdin PRS	0.544	0.468	0.466	0.476
Chen PRS	0.002	0	0	0
Vujkovic PRS	0.002	0	0	0.004
Ghouse PRS	0.006	0	0	0
Whitfield PRS	0.002	0	0	0
Children (*n* = 899)				
Chen PRS	0.922	0.926	0.93	0.926
Ghouse PRS	0.06	0.052	0.052	0.056
Whitfield PRS	0.006	0.006	0.002	0.002
Schwantes‐An PRS	0.012	0.016	0.016	0.016
NAS ≥ 4				
Adult (*n* = 2149)				
Chen PRS	1	1	1	1
Children (*n* = 900)				
Schwantes‐An PRS	0.504	0.628	0.628	0.694
Chen PRS	0.19	0.176	0.194	0.154
Whitfield PRS	0.262	0.096	0.088	0.05
Vujkovic PRS	0.042	0.1	0.09	0.102
Emdin PRS	0.002	0	0	0

^a^ Adjusted for ancestry, age, and sex.

### 3.6 Relationship Between Top PRSs Quartiles and Key MASH Histological Lesions

In children and adults, the Chen and Schwantes‐An PRSs were associated with a significant increase in the odds of steatosis and lobular inflammation but not hepatocellular ballooning (Table 4).

**TABLE 4:** Association of top polygenic risk scores with cardinal MASH histological lesions.

PRS	Trait	Adult			Children		
		Odds ratio	95% CI	*p*	Odds ratio	95% CI	*p*
Chen	Steatosis (Grade ≥ 2 vs. < 2)	1.29	(1.18–1.40)	1.28E‐08	1.57	(1.33–1.85)	7.47E‐08
	Lobular Inflammation (Grade ≥ 2 vs. < 2)	1.19	(1.10–1.30)	4.42E‐05	1.22	(1.06–1.42)	6.99E‐03
	Ballooning (Grade ≥ 1 vs. 0)	1.1	(1.00–1.20)	4.24E‐02	0.96	(0.82–1.11)	5.63E‐01
Schwantes‐An	Steatosis (Grade ≥ 2 vs. < 2)	1.13	(1.04–1.23)	5.53E‐03	1.48	(1.27–1.74)	1.09E‐06
	Lobular Inflammation (Grade ≥ 2 vs. < 2)	1.22	(1.12–1.33)	4.45E‐06	1.17	(1.02–1.35)	2.74E‐02
	Ballooning (Grade ≥ 1 vs. 0)	1.04	(0.95–1.13)	4.22E‐01	0.9	(0.78–1.04)	1.65E‐01

*Note:* Data shown for all participants and is adjusted for ancestry, age, and sex.

Compared to participants in the bottom quartile of the Chen PRS, those in the top quartile also had significantly higher odds of severe steatosis, severe lobular inflammation, NAS ≥ 4, advanced fibrosis, and at‐risk MASH (Figure 2; Table 5). Similar associations were observed for those in the top quartile of the Schwantes‐An PRS but mainly in adults (Figure 2; Table 5).

**FIGURE 2:**
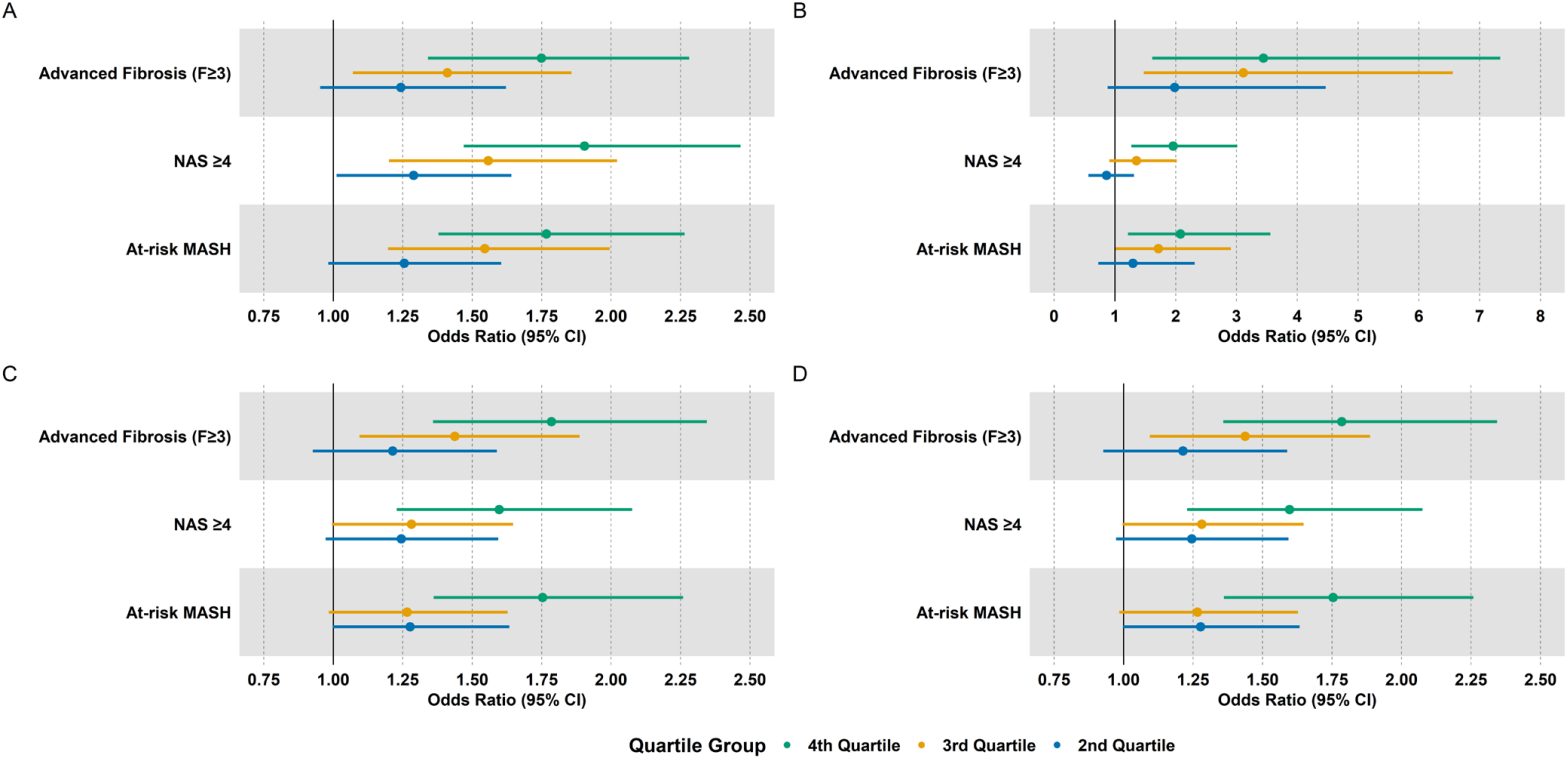
Association of Chen and Schwantes‐An polygenic risk score (PRS) quartiles with key MASLD histological phenotypes. Panels show the associations of the Chen PRS in (A) adults and (B) children, and the Schwantes‐An PRS in (C) adults and (D) children. Odds ratios (ORs) and 95% confidence intervals are shown for the 2nd, 3rd, and 4th PRS quartiles relative to the lowest quartile (reference group). Individuals in the highest quartile of the Chen PRS had increased odds of NAS ≥ 4, at‐risk MASH, and advanced fibrosis in both adults and children. Similarly, the highest quartile of the Schwantes‐An PRS was associated with all three outcomes in adults, whereas in children it was significantly associated with NAS ≥ 4 but not with at‐risk MASH or advanced fibrosis. Data shown for all participants and is adjusted for ancestry, age, and sex.

**TABLE 5:** Risk of key MASLD phenotypes per Chen and Schwantes‐An PRS quartiles.

Trait	Age group	Category (1st quartile as reference)	Chen			Schwantes‐An		
			Odds ratio	95% CI	*p*	Odds ratio	95% CI	*p*
Steatosis (Grade ≥ 2)	Adult	4th Quartile	1.78	(1.39–2.27)	3.90E‐06	1.35	(1.06–1.73)	1.69E‐02
		3rd Quartile	1.43	(1.11–1.83)	4.97E‐03	1.26	(0.99–1.60)	6.31E‐02
		2nd Quartile	1.12	(0.89–1.42)	3.26E‐01	1.03	(0.81–1.31)	8.04E‐01
	Children	4th Quartile	3.23	(2.01–5.18)	1.27E‐06	3.74	(2.43–5.76)	2.25E‐09
		3rd Quartile	1.74	(1.15–2.63)	9.19E‐03	1.97	(1.31–2.96)	1.09E‐03
		2nd Quartile	1.12	(0.72–1.73)	6.23E‐01	2.59	(1.67–4.02)	1.96E‐05
Lobular inflammation (Grade ≥ 2)	Adult	4th Quartile	1.59	(1.25–2.02)	1.67E‐04	1.61	(1.26–2.06)	1.46E‐04
		3rd Quartile	1.56	(1.22–2.00)	3.93E‐04	1.52	(1.19–1.93)	6.95E‐04
		2nd Quartile	1.49	(1.17–1.88)	9.82E‐04	1.53	(1.20–1.94)	4.80E‐04
	Children	4th Quartile	1.64	(1.08–2.49)	2.05E‐02	1.87	(1.26–2.79)	2.07E‐03
		3rd Quartile	1.39	(0.93–2.08)	1.08E‐01	1.52	(1.01–2.27)	4.48E‐02
		2nd Quartile	1.2	(0.77–1.85)	4.20E‐01	1.85	(1.22–2.81)	3.87E‐03
Ballooning (Grade ≥ 1)	Adult	4th Quartile	1.27	(0.99–1.63)	6.05E‐02	1.14	(0.88–1.47)	3.28E‐01
		3rd Quartile	1.13	(0.88–1.46)	3.45E‐01	0.93	(0.72–1.19)	5.42E‐01
		2nd Quartile	1.08	(0.85–1.38)	5.30E‐01	1.1	(0.86–1.41)	4.63E‐01
	Children	4th Quartile	0.85	(0.56–1.30)	4.58E‐01	0.97	(0.65–1.44)	8.79E‐01
		3rd Quartile	0.91	(0.61–1.36)	6.33E‐01	0.78	(0.52–1.16)	2.20E‐01
		2nd Quartile	0.89	(0.58–1.38)	6.05E‐01	1.04	(0.69–1.58)	8.41E‐01
NAS ≥ 4	Adult	4th Quartile	1.9	(1.47–2.47)	1.07E‐06	1.6	(1.23–2.08)	4.68E‐04
		3rd Quartile	1.56	(1.20–2.02)	8.56E‐04	1.28	(1.00–1.65)	5.37E‐02
		2nd Quartile	1.29	(1.01–1.64)	3.96E‐02	1.24	(0.97–1.59)	8.17E‐02
	Children	4th Quartile	1.96	(1.27–3.01)	2.33E‐03	3.02	(2.00–4.54)	1.24E‐07
		3rd Quartile	1.35	(0.91–2.02)	1.39E‐01	1.43	(0.97–2.12)	7.05E‐02
		2nd Quartile	0.86	(0.56–1.31)	4.85E‐01	1.69	(1.12–2.55)	1.17E‐02
Advanced fibrosis	Adult	4th Quartile	1.75	(1.34–2.28)	3.70E‐05	1.78	(1.36–2.34)	3.11E‐05
		3rd Quartile	1.41	(1.07–1.86)	1.44E‐02	1.44	(1.09–1.89)	9.02E‐03
		2nd Quartile	1.24	(0.95–1.62)	1.09E‐01	1.21	(0.93–1.59)	1.60E‐01
	Children	4th Quartile	3.44	(1.61–7.34)	1.38E‐03	1.79	(0.97–3.29)	6.04E‐02
		3rd Quartile	3.11	(1.48–6.56)	2.84E‐03	2.16	(1.18–3.96)	1.26E‐02
		2nd Quartile	1.98	(0.88–4.47)	9.94E‐02	1.1	(0.56–2.18)	7.79E‐01
At‐risk MASH	Adult	4th Quartile	1.77	(1.38–2.26)	6.93E‐06	1.75	(1.36–2.26)	1.38E‐05
		3rd Quartile	1.55	(1.20–2.00)	8.46E‐04	1.27	(0.98–1.63)	6.66E‐02
		2nd Quartile	1.26	(0.98–1.60)	6.94E‐02	1.28	(1.00–1.63)	5.23E‐02
	Children	4th Quartile	2.08	(1.21–3.56)	7.69E‐03	1.35	(0.84–2.16)	2.21E‐01
		3rd Quartile	1.71	(1.01–2.91)	4.59E‐02	1.21	(0.74–1.97)	4.39E‐01
		2nd Quartile	1.3	(0.73–2.31)	3.79E‐01	1.08	(0.65–1.80)	7.77E‐01

*Note:* Data shown for all participants and is adjusted for ancestry, age, and sex.

### 3.7 Diagnostic Performance of PRSs for Detecting Advanced Fibrosis and NAS ≥ 4

In adults, the Schwantes‐An PRS had weaker diagnostic performance for detecting advanced fibrosis than FIB‐4's (AUROC 0.563 vs. 0.789, *p* < 0.001, Figure 3A). In children, there was no significant difference between the Chen PRS and FIB‐4 AUROCs (0.6 vs. 0.612, *p* > 0.05, Figure 3B; Table S7). In adults and children, adding the PRS to FIB‐4 with additional clinical risk factors such as demographic covariates (age, sex, and ancestry), obesity, and diabetes did not significantly improve FIB‐4's performance for predicting advanced fibrosis (Table S7).

**FIGURE 3:**
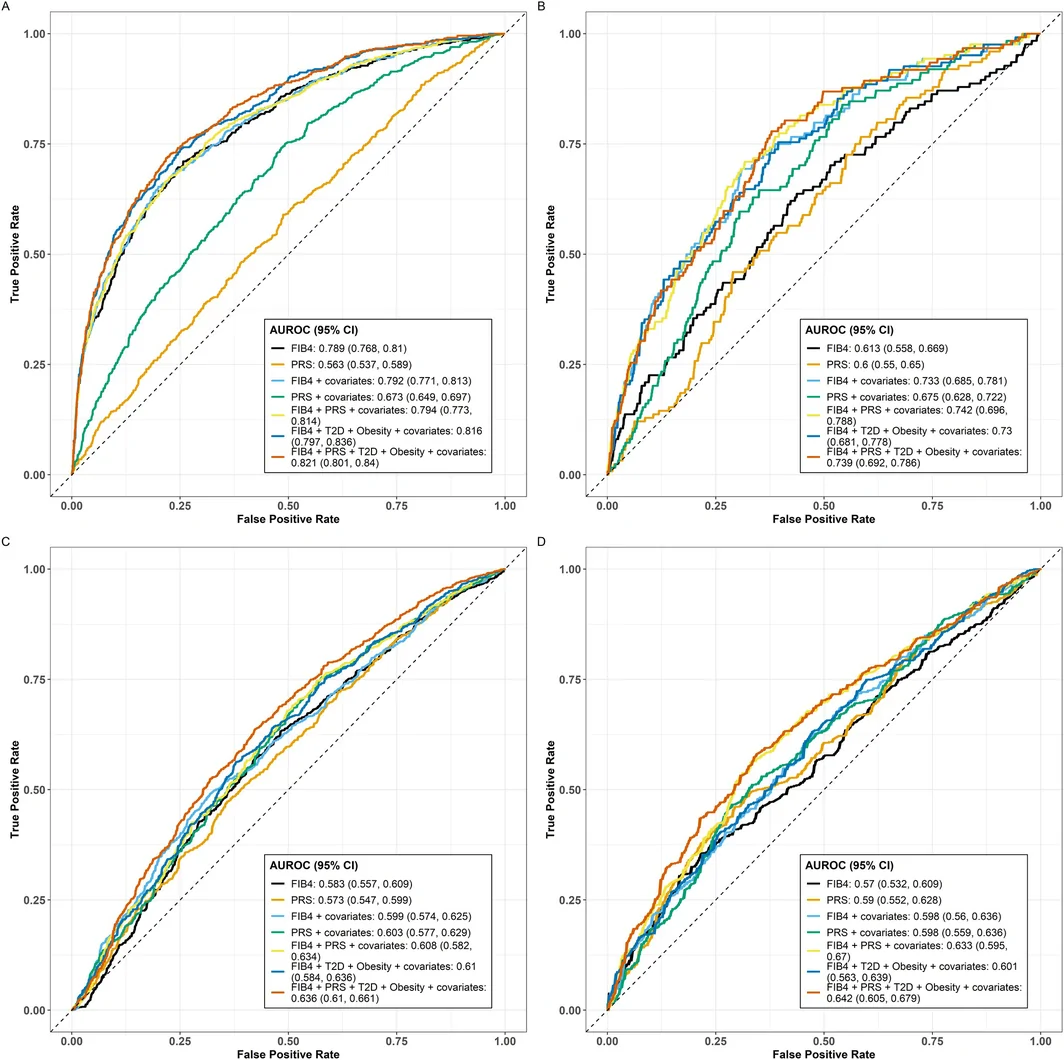
Diagnostic performance of polygenic risk scores in comparison to other models for predicting advanced fibrosis in (A) Adults using Schwantes‐An PRS, and in (B) children using Chen PRS; and for predicting NAS ≥ 4 in (C) Adults using Chen PRS, and in (D) children using Schwantes‐An PRS. AUROC and 95% CI for each model are listed in the legend. Model 1: FIB4, Model 2: PRS, Model 3: Outcome ~ FIB4 + demographic covariates (ancestry + age + sex), Model 4: Outcome ~ PRS + demographic covariates, Model 5: Outcome ~ FIB4 + PRS + demographic covariates, Model 6: Outcome ~ FIB4 + Type 2 diabetes + obesity + demographic covariates, Model 7: Outcome ~ FIB4 + PRS + Type 2 diabetes + obesity + demographic covariates.

In adults and children, the Chen and Schwantes‐An PRSs and FIB‐4 showed poor discrimination ability (AUROC < 0.60) for NAS ≥ 4 (*p* > 0.05; Figure 3C,D). In children, adding the PRS to a model containing FIB‐4 and demographic covariates significantly improved its ability to predict NAS ≥ 4 (AUROC 0.598 vs. 0.633, *p* = 0.02, Figure 3D; Table S7). In both adults and children, adding the PRSs to a model that included FIB‐4, demographic covariates, obesity, and diabetes significantly improved the AUROC for predicting NAS ≥ 4 (AUROC adults 0.61 vs. 0.636, children 0.601 vs. 0.642, *p* < 0.05 for both, Figure 3C,D; Table S7) but the discrimination ability of these models for NAS ≥ 4 remained modest.

## 4 Discussion

This study examined the association of several PRSs with MASLD histology in a large histologically phenotyped cohort of adults and children. Using a penalized regression process with several selection methods that included all PRSs simultaneously, the Schwantes‐An and Emdin PRSs emerged as the leading PRSs in adults for advanced fibrosis, with the Schwantes‐An PRS being selected by most penalized models; whereas in children, the Chen PRS was selected most frequently. For NAS ≥ 4, the Chen PRS in adults and the Schwantes‐An PRS in children were consistently selected. Overall, individuals in the top quartiles of the Chen and Schwantes‐An PRSs had higher risks for MASLD histological phenotypes than those in their bottom quartiles. Neither PRS showed good diagnostic performance for predicting advanced fibrosis or NAS ≥ 4 in children or adults with MASLD.

Association of candidate gene variants or combinations of them was shown in studies of adults with MASLD to be associated with the risk of MASLD, its histological activity and severity, or its complications [15, 18, 20, 29, 42, 43]. Several small studies evaluated the association of candidate gene‐based genetic risk scores with MASLD and its histology in children [19, 44, 45, 46]. In the larger Genetics of Obesity Associated Liver Steatosis (GOALS) study in children [34], the association of 60 variants of candidate genes with MASLD and fibrosis was assessed in 822 children who were part of the current study. The GOALS study reported the association of variants in *SLC27A5*, *SAMM50*, *PARVB* (rs6006473), *LYPLAL1*, *PNPLA3* (rs6006460), *TNF*, *APOE* and *SLC2A1* with MASLD risk and *PARVB* (rs6006473) and *ADIPOR2* with fibrosis risk in children. However, the association of recently described GWAS‐derived PRSs with MASLD histology in adults and children has not been previously characterized.

Despite the significant correlation observed in this study among the seven examined PRSs which suggests shared genetic architecture among PRSs, the penalized regression models selected the 17 SNP Chen PRS as the lead PRS associated with MASLD disease activity in adults and with advanced fibrosis in children, suggesting age‐specific effects for this PRS on these traits. This PRS was derived and validated using a multiancestry GWAS meta‐analysis of multiple cohorts with imaging‐measured hepatic steatosis and diagnostic codes‐diagnosed MASLD [22]. Our data validate this PRS association with MASLD and show it is also associated with the risk of MASLD histological activity and severity. In addition to novel MASLD‐associated variants, the Chen PRS includes several well‐validated genetic variants associated with MASLD and its histological activity and severity including variants in *PNPLA3*, *TM6SF2*, *GCKR*, and TMC4/*MBOAT7* [47].

The Schwantes‐An PRS, on the other hand, was the lead PRS selected by most penalized regression models for association with advanced fibrosis in adults and MASLD activity in children, suggesting age‐specific effects for this PRS on these traits. This 20 SNP PRS was derived and validated using meta‐analysis of GWAS of several cohorts with ALD cirrhosis and includes novel variants in addition to established MASLD‐associated variants like those in *PNPLA3, MBOAT7, HSD17B13* [39].

We observed no interaction between PRSs and age or sex for the risk of NAS ≥ 4 in EUR or AMR participants, but several PRSs interacted with sex in relation to advanced fibrosis among participants of European ancestry. Given the multiple interactions tested without correction for multiple comparisons, these analyses are considered exploratory and the findings will require further study.

Risk stratification of individuals with MASLD based on their genetic susceptibility is a potential benefit of PRSs [24]. Overall, the risk of MASLD histological traits increased with increasing Chen and Schwantes‐An PRS in both age groups, but the magnitude of risk increase was more pronounced in children. Compared to those in the bottom quartiles, children in the top quartile of the Chen score had 3.5‐fold of advanced fibrosis and almost 2‐fold higher risk of having NAS ≥ 4 or at‐risk MASH, whereas adults in the top quartile of Chen PRS had 62%, 91%, 53% increase in the risks of advanced fibrosis, NAS ≥ 4, and at risk MASH, respectively versus those in the lowest quartile. Similarly, adults in the top quartile of Schwantes‐An PRS had 71%, 63%, 69%, respectively, for these primary phenotypes versus those in the bottom quartile. The findings suggest genetic susceptibility is a more prominent determinant of MASLD histological activity and severity in childhood but these effects taper off in adulthood likely due to non‐genetic factors such as metabolic factors, alcohol consumption, diet quality, and physical activity [48]. However, the observed increased risk of these traits in association with high PRSs did not translate into good PRS clinical discrimination of advanced fibrosis or NAS ≥ 4. Adding a PRS to FIB‐4, with or without additional clinical risk factors (age, sex, ancestry, obesity, and diabetes), did not significantly improve FIB‐4's performance for detecting advanced fibrosis. This finding likely reflects the fundamentally different information captured by these measures. PRSs estimate inherited susceptibility to developing disease, whereas FIB‐4 is designed to detect established fibrosis at a given point in time. As a result, genetic risk does not necessarily translate into improved diagnostic or prognostic performance once the disease is present. This phenomenon has been described in other disease states such as coronary artery disease (CAD), where a PRS does not outperform or improve the detection of CAD compared to other clinical prediction tools or biomarkers [49, 50]. Genetic susceptibility represents only one component of MASLD risk and progression, which are also strongly influenced by metabolic factors, lifestyle exposures, and aging. Consequently, a fibrosis biomarker such as FIB‐4, which directly reflects the downstream consequences of chronic liver injury, is more proximal to advanced fibrosis than a PRS, which acts upstream in the disease pathway. These findings suggest that PRSs are likely to be most useful for identifying individuals at increased risk before advanced disease develops or in settings where phenotype‐specific biomarkers are not available.

The study has several strengths. The cohort is the largest with histologically phenotyped MASLD in adults and children. Histology was systematically and centrally assessed by the NASH CRN Pathology Committee on all included subjects. The study is also the first to examine in depth the associations of recently described liver disease GWAS‐derived PRSs with MASLD histology in adults and children.

This study also has limitations. Most of the adults included in this study were White females and most of the children were Hispanic males, thus it is unclear if the findings could be fully extended to other patients with MASLD from different demographic profiles in the corresponding age group. The study did not report on the association of the PRS with clinical outcomes since MASLD histology, and not clinical outcomes, was the focus of this analysis.

In summary, this study shows two model‐selected PRSs (Chen and Schwantes‐An) are associated with advanced fibrosis and histological activity in MASLD. Although both PRSs were associated with higher MASLD severity, neither demonstrated good diagnostic performance for detecting advanced fibrosis or NAS ≥ 4. Our findings suggest the current PRSs may be better suited as risk‐stratification rather than diagnostic tools for advanced MASLD. Further studies are needed to determine whether PRSs alone or in combination with non‐invasive fibrosis tests and clinical prediction models add value in predicting MASLD progression or long‐term outcomes.

## Author Contributions

Design: S.G., X.G., J.I.R., N.C. Analysis: J.T., X.G. Manuscript writing: S.G. Manuscript review and revision: all authors.

## Funding

This work was also supported in part by the NIDDK Nonalcoholic Steatohepatitis Clinical Research Network (NASH CRN) grants (U01DK061730, U24DK061730, U01DK061737), the National Center for Advancing Translational Sciences, CTSI grants (UL1TR001881, UL1TR000006), the NIDDK Diabetes Research Center (DRC) grant DK063491 to the Southern California Diabetes Endocrinology Research Center. This work is also supported by the Intramural Research Program of the National Institutes of Health, National Cancer Institute. Infrastructure for the CHARGE Consortium is supported in part by the National Heart, Lung, and Blood Institute (NHLBI) grant R01HL105756. Authors would also like to acknowledge Luca Lotta and Niek Verweij from Regeneron Genetics Center for their help with the whole‐exome sequencing and genotyping work used in this study.

## Conflicts of Interest

Authors declare none for this paper. For full disclosure, the authors share the following information: Samer Gawrieh: provided consulting services to TransMedics, Kowa, Spruce, and Novo Nordisk, and received research grant support from DSM and Zydus. Jeffrey B. Schwimmer consults for Merck and receives grants from Intercept and Seraphina. Naga P. Chalasani has ongoing or recent (within 12 months) consulting agreements with Madrigal, Zydus, BioMea, GSK, Chugai, Eccogene Ipsen, and Altimmune. He receives research support from Exact Sciences, Boehringer Ingelheim, and Madrigal. He has equity interest in Avant Sante Inc. (a contract research organization) and Heligenics (a drug discovery startup). These relationships are not significantly or directly related to this paper. The remaining authors have no conflicts to report.

## Supporting information


**Table S1:** Polygenic risk scores examined in this study.
**Table S2:** Polygenic scores with missing single nucleotide polymorphisms.
**Table S3:** Participants characteristics.
**Table S4:** Interaction analysis of PRS with age and sex on advanced fibrosis by ancestry.
**Table S5:** Interaction analysis of PRS with age and sex on NAS ≥ 4 by ancestry.
**Table S6:** Sensitivity analysis for frequency of the top selected PRS for advanced fibrosis and NAS ≥ 4 in dominant ancestry in each age group.
**Table S7:** Comparison of diagnostic performance of FIB‐4, polygenic risk score and alone or in combination with other clinical variables for detection of advanced fibrosis or NAS ≥ 4.
**Figure S1:** Nomograms of polygenic risk scores distributions in the cohort. Dashed vertical line represents the mean. Overall, children had slightly higher mean PRSs compared to adults.

## Data Availability

Data may be made available upon special request to the authors.
