# Supplementary material for: Age‐Specific Associations of Polygenic Risk Scores With Advanced Fibrosis and Histological Activity in Biopsy‐Proven MASLD
Source: Liver Int. 2026 Oct 1;46(11):e70907. doi: 10.1111/liv.70907 (PMC13629459; doi:10.1111/liv.70907)
Supplement: Supplementary file 1 [file LIV-46-0-s001.zip › liv70907-sup-0001-Supinfo1@PRS Supplemental- 8.24.26.docx]

**Supplemental Material**

**Age-specific Associations of Polygenic Risk Scores with Advanced Fibrosis and Histological Activity in Biopsy-proven MASLD**

Samer Gawrieh^1*^, Jingyi Tan^2*^, Xiuqing Guo^2^, Linus Schwantes-An^3^, Marco Abreu^3^, Callie J. Zaborenko^3^, Eduardo Vilar-Gomez^1^, Jeffrey B. Schwimmer^4^, Jerome I. Rotter^2^, Naga Chalasani^1^

**Supplemental Table 1. Polygenic risk scores examined in this study**

| **PRS** | **Authors, Year** | **Number of SNPs** | **Races/ethnicities** | **Disease/Condition** |
| --- | --- | --- | --- | --- |
| Chen | Chen et al., 2023 | 17 | multi-ancestry | MASLD defined by imaging or diagnostic codes |
| Ghouse | Ghouse et al., 2024 | 36 | multi-ancestry | Cirrhosis defined by diagnostic codes |
| Emdin | Emdin et al., 2021 | 12 | multi-ancestry | Cirrhosis defined by diagnostic codes, or clinical, laboratory, histology or imaging, or self-reported. |
| Schwantes-An | Schwantes-An et al., 2024 | 20 | European | Alcohol-associated cirrhosis defined by diagnostic codes |
| Vujkovic | Vujkovic et al., 2022 | 77 | multi-ancestry | MASLD by chronic ALT, imaging and histology |
| Vujkovic 17 | Vujkovic et al., 2022 | 17 | multi-ancestry | MASLD as above |
| Whitfield | Whitfield et al., 2022 | 3 | European | Alcohol-associated cirrhosis defined by diagnostic codes, or clinical, laboratory, histology or imaging |

Abbreviations: PRS: polygenic risk score, SNPs: Single Nucleotide Polymorphisms.

**Supplemental Table 2. Polygenic scores with missing single nucleotide polymorphisms**

| **PRS** | **Authors, Year** | **Number of SNPs** | **Missing SNPs** | **RS missing SNP** |
| --- | --- | --- | --- | --- |
| Chen | Chen et al., 2023 | 17 | 2 | rs10756038  rs140201358 |
| Ghouse | Ghouse et al., 2024 | 36 | 2 | rs146650659  rs113469203 |
| Emdin | Emdin et al., 2021 | 12 | 0 |  |
| Schwantes-An | Schwantes-An et al., 2024 | 20 | 2 | rs13055235  rs199571141 |
| Vujkovic | Vujkovic et al., 2022 | 77 | 3 | rs574044675  rs74816838  rs141505249 |
| Vujkovic 17 | Vujkovic et al., 2022 | 17 | 0 |  |
| Whitfield | Whitfield et al., 2022 | 3 | 0 |  |

SNP: Single nucleotide polymorphisms.

**Supplemental Table 3. Participants characteristics**

| **Variables** | **Adults (EUR/AMR*)**  **N=2149** | **Children**  **(EUR/AMR*)**  **N=900** |
| --- | --- | --- |
| Age, years (Mean, IQR) | 50.24 (16.56) | 12.88 (4.24) |
| Male (n, %) | 798 (37.13%) | 657 (73%) |
| White (n, %) | 1791 (83.34%) | 219 (24.33%) |
| Hispanic/Latino ethnicity (n, %) | 358 (16.66%) | 681 (75.67%) |
| Waist circumference, cm (Mean, SD) | 109.97 (14.4) | 103.82 (15.29) |
| BMI, kg/m2 (Mean, SD) | 34.62 (6.47) | 32.1 (6.31) |
| Obesity, BMI ≥ 30 kg/m^2^ (n, %) | 1596 (74.27%) | 530 (58.89%) |
| Type2 diabetes mellitus (n, %) | 789 (36.71%) | 33 (3.67%) |
| **Liver histology** |  |  |
| Steatosis grade |  |  |
| <5% (0) (n, %) | 120 (5.58%) | 28 (3.11%) |
| 5-33% (1) (n, %) | 801 (37.27%) | 228 (25.33%) |
| 34-66% (2) (n, %) | 689 (32.06%) | 260 (28.89%) |
| >66% (3) (n, %) | 539 (25.08%) | 384 (42.67%) |
| Lobular inflammation |  |  |
| None (0) (n, %) | 22 (1.02%) | 4 (0.44%) |
| <2 (1) (n, %) | 1150 (53.51%) | 515 (57.22%) |
| 2-4 (2) (n, %) | 751 (34.95%) | 323 (35.89%) |
| >4 (3) (n, %) | 226 (10.52%) | 58 (6.44%) |
| Ballooning degeneration |  |  |
| None (0) (n, %) | 764 (35.55%) | 575 (63.89%) |
| Few (1) (n, %) | 602 (28.01%) | 222 (24.67%) |
| Many (2) (n, %) | 783 (36.44%) | 103 (11.44%) |
| NAFLD Activity score (NAS) (Mean, SD) | 4.32 (1.71) | 4.07 (1.52) |
| NAS ≥ 4 (n, %) | 1451 (67.52%) | 579 (64.33%) |
| MASH diagnosis |  |  |
| MASLD, not MASH (0) (n, %) | 435 (20.24%) | 257 (28.56%) |
| Borderline MASH (1) (n, %) | 408 (18.99%) | 422 (46.89%) |
| Definite MASH (2) (n, %) | 1233 (57.38%) | 195 (21.67%) |
| At-risk MASH (n, %) | 859 (39.97%) | 187 (20.78%) |
| Fibrosis stage |  |  |
| None (F0) (n, %) | 520 (24.2%) | 299 (33.22%) |
| Mild/Moderate (1a, 1b, 1c)= (F1) (n, %) | 542 (25.22%) | 356 (39.56%) |
| Zone 3 and Periportal (F2) (n, %) | 415 (19.31%) | 120 (13.33%) |
| Bridging (F3) (n, %) | 438 (20.38%) | 112 (12.44%) |
| Cirrhosis (F4) (n, %) | 225 (10.47%) | 12 (1.33%) |

* Genetic ancestry confirmed by SNPRelate; EUR: European ancestry, AMR: Amerindian ancestry (Hispanic).

**Supplemental Table 4. Interaction analysis of PRS with age and sex on advanced fibrosis by ancestry**

|  |  |  | EUR | | AMR | |
| --- | --- | --- | --- | --- | --- | --- |
| Trait | PRS | Interaction Term | OR_95CI | p.value | OR_95CI | p.value |
| Advanced Fibrosis | Chen PRS | PRS * age | 1.01 (1.00-1.02) | 1.06E-01 | 0.99 (0.97-1.02) | 6.40E-01 |
| Advanced Fibrosis | Chen PRS | PRS * sex | 1.31 (1.05-1.64) | 1.95E-02 | 1.02 (0.60-1.78) | 9.31E-01 |
| Advanced Fibrosis | Emdin PRS | PRS * age | 1.00 (0.99-1.01) | 4.50E-01 | 1.00 (0.98-1.02) | 7.91E-01 |
| Advanced Fibrosis | Emdin PRS | PRS * sex | 1.32 (1.05-1.66) | 1.75E-02 | 0.96 (0.57-1.60) | 8.66E-01 |
| Advanced Fibrosis | Schwantes-An PRS | PRS * age | 1.01 (1.00-1.02) | 2.39E-01 | 1.00 (0.98-1.02) | 9.07E-01 |
| Advanced Fibrosis | Schwantes-An PRS | PRS * sex | 1.06 (0.85-1.33) | 6.12E-01 | 0.95 (0.54-1.70) | 8.66E-01 |
| Advanced Fibrosis | Whitfield PRS | PRS * age | 1.01 (1.00-1.02) | 1.05E-01 | 0.99 (0.97-1.02) | 6.17E-01 |
| Advanced Fibrosis | Whitfield PRS | PRS * sex | 1.20 (0.96-1.51) | 1.10E-01 | 0.80 (0.46-1.40) | 4.20E-01 |
| Advanced Fibrosis | Vujkovic PRS | PRS * age | 1.01 (1.00-1.02) | 2.28E-01 | 0.99 (0.97-1.02) | 6.59E-01 |
| Advanced Fibrosis | Vujkovic PRS | PRS * sex | 1.23 (0.98-1.54) | 7.52E-02 | 0.65 (0.35-1.17) | 1.54E-01 |
| Advanced Fibrosis | Ghouse PRS | PRS * age | 1.00 (0.99-1.01) | 4.61E-01 | 1.00 (0.98-1.02) | 7.84E-01 |
| Advanced Fibrosis | Ghouse PRS | PRS * sex | 1.17 (0.93-1.46) | 1.75E-01 | 0.85 (0.49-1.47) | 5.56E-01 |
| Advanced Fibrosis | Vujkovic17 PRS | PRS * age | 1.00 (0.99-1.01) | 4.64E-01 | 1.00 (0.98-1.03) | 9.19E-01 |
| Advanced Fibrosis | Vujkovic17 PRS | PRS * sex | 1.35 (1.08-1.69) | 8.99E-03 | 1.06 (0.59-1.91) | 8.47E-01 |

**Supplemental Table 5. Interaction analysis of PRS with age and sex on NAS≥4 by ancestry**

|  |  |  | EUR | | AMR | |
| --- | --- | --- | --- | --- | --- | --- |
| Trait | PRS | Interaction Term | OR_95CI | p.value | OR_95CI | p.value |
| NAS ≥ 4 | Chen PRS | PRS * age | 1.00 (0.99-1.01) | 4.13E-01 | 0.99 (0.97-1.01) | 2.23E-01 |
| NAS ≥ 4 | Chen PRS | PRS * sex | 1.13 (0.92-1.40) | 2.50E-01 | 0.72 (0.47-1.10) | 1.27E-01 |
| NAS ≥ 4 | Emdin PRS | PRS * age | 1.01 (1.00-1.02) | 1.57E-01 | 0.99 (0.98-1.01) | 5.22E-01 |
| NAS ≥ 4 | Emdin PRS | PRS * sex | 0.91 (0.73-1.12) | 3.69E-01 | 0.82 (0.54-1.23) | 3.28E-01 |
| NAS ≥ 4 | Schwantes-An PRS | PRS * age | 1.01 (1.00-1.02) | 1.28E-01 | 1.00 (0.99-1.02) | 6.04E-01 |
| NAS ≥ 4 | Schwantes-An PRS | PRS * sex | 0.86 (0.70-1.07) | 1.70E-01 | 0.79 (0.52-1.22) | 2.87E-01 |
| NAS ≥ 4 | Whitfield PRS | PRS * age | 1.01 (1.00-1.02) | 5.04E-02 | 1.00 (0.98-1.02) | 9.76E-01 |
| NAS ≥ 4 | Whitfield PRS | PRS * sex | 0.99 (0.80-1.22) | 8.91E-01 | 0.77 (0.51-1.16) | 2.17E-01 |
| NAS ≥ 4 | Vujkovic PRS | PRS * age | 0.99 (0.99-1.00) | 2.79E-01 | 0.99 (0.97-1.01) | 4.13E-01 |
| NAS ≥ 4 | Vujkovic PRS | PRS * sex | 1.07 (0.87-1.33) | 5.24E-01 | 0.91 (0.57-1.45) | 6.83E-01 |
| NAS ≥ 4 | Ghouse PRS | PRS * age | 1.00 (0.99-1.01) | 9.77E-01 | 1.00 (0.98-1.01) | 7.38E-01 |
| NAS ≥ 4 | Ghouse PRS | PRS * sex | 1.01 (0.82-1.25) | 9.35E-01 | 0.83 (0.55-1.26) | 3.78E-01 |
| NAS ≥ 4 | Vujkovic17 PRS | PRS * age | 1.00 (0.99-1.01) | 8.42E-01 | 0.99 (0.97-1.01) | 3.91E-01 |
| NAS ≥ 4 | Vujkovic17 PRS | PRS * sex | 1.00 (0.81-1.24) | 9.80E-01 | 0.74 (0.46-1.18) | 2.04E-01 |

**Supplemental Table 6. Sensitivity analysis for frequency of the top selected PRS for advanced fibrosis and NAS ≥ 4 in dominant ancestry in each age group**

| **Train/Group/PRS** | **Stepwise AIC** | **LASSO** | **Elastic-Net** | **Ridge** |
| --- | --- | --- | --- | --- |
| Advance fibrosis |  |  |  |  |
| Adult EUR  (n = 1784) |  |  |  |  |
| Emdin PRS | 0.558 | 0.444 | 0.44 | 0.468 |
| Schwantes-An PRS | 0.43 | 0.544 | 0.554 | 0.528 |
| Whitfield PRS | 0.006 | 0.012 | 0.006 | 0.004 |
| Vujkovic PRS | 0.006 | 0 | 0 | 0 |
| Children  AMR (n = 681) |  |  |  |  |
| Chen PRS | 0.936 | 0.936 | 0.932 | 0.94 |
| Whitfield PRS | 0 | 0.006 | 0.012 | 0.006 |
| Ghouse PRS | 0.022 | 0.02 | 0.018 | 0.018 |
| Schwantes-An PRS | 0.018 | 0.026 | 0.026 | 0.026 |
| Emdin PRS | 0.022 | 0.012 | 0.012 | 0.01 |
| **NAS ≥4** |  |  |  |  |
| Adult  EUR (n = 1791) |  |  |  |  |
| Chen PRS | 0.982 | 0.984 | 0.984 | 0.986 |
| Emdin PRS | 0.014 | 0.012 | 0.012 | 0.012 |
| Whitfield PRS | 0.004 | 0.004 | 0.004 | 0.002 |
| Children  AMR (n = 681) |  |  |  |  |
| Schwantes-An PRS | 0.746 | 0.91 | 0.888 | 0.922 |
| Whitfield PRS | 0.212 | 0.058 | 0.072 | 0.036 |
| Emdin PRS | 0.022 | 0.01 | 0.01 | 0.006 |
| Vujkovic PRS | 0.018 | 0.022 | 0.028 | 0.036 |
| Chen PRS | 0 | 0 | 0.002 | 0 |
| Ghouse PRS | 0.002 | 0 | 0 | 0 |

**Supplemental Table 7. Comparison of diagnostic performance of FIB-4, polygenic risk score and alone or in combination with other clinical variables for detection of advanced fibrosis or NAS ≥ 4**

|  |  | **Advanced Fibrosis** | | **NAS ≥ 4** | |
| --- | --- | --- | --- | --- | --- |
| Model1 | Model2 | Adult (Schwantes-An PRS) | Children (Chen PRS) | Adult (Chen PRS) | Children (Schwantes-An PRS) |
| FIB4 | PRS | 2.04E-41 | 7.18E-01 | 5.97E-01 | 4.63E-01 |
| FIB4 + demographic covariates* | FIB4 + PRS + demographic covariates | 4.65E-01 | 2.71E-01 | 3.78E-01 | 2.01E-02 |
| FIB4 + T2D + Obesity + demographic covariates | FIB4 + PRS + T2D + Obesity + demographic covariates | 9.96E-02 | 2.44E-01 | 1.67E-03 | 6.75E-03 |

* Demographic covariates include ancestry + age + sex

**Figure legends**

**Supplemental Figure 1. Nomograms of polygenic risk scores distributions in the cohort.** Dashed vertical line represents the mean. Overall, children had slightly higher mean PRSs compared to adults.
